# Intelligent Robot-Aided, Task-Oriented Physiotherapy Method for Upper Limb Rehabilitation: Development and Usability Study

**DOI:** 10.2196/92898

**Published:** 2026-09-01

**Authors:** Piotr Falkowski, Kajetan Jeznach, Jan Oleksiuk, Tomasz Osiak, Maciej Pikuliński, Andrzej Zakręcki, Krzysztof Zawalski, Piotr Kołodziejski, Daniel Śliż

**Affiliations:** 1 Section of Biorobotics and Medical Devices, Łukasiewicz Research Network—Industrial Research Institute for Automation and Measurements PIAP, Al Jerozolimskie 202, Warsaw, Mazovia, 02-486, Poland, 48 228740340; 2 Institute of Aeronautics and Applied Mechanics, Faculty of Power and Aeronautical Engineering, Warsaw University of Technology, Warsaw, Poland; 3 Department of Manufacturing Systems, Faculty of Mechanical Engineering and Robotics, AGH University of Science and Technology in Cracow, Krakow, Poland; 4 3rd Department of Internal Diseases and Cardiology, Medical University of Warsaw, Warsaw, Poland

**Keywords:** electromyography, exoskeleton, home therapy, intelligent algorithms, minimally supervised treatment, personalized medicine, rehabilitation robotics

## Abstract

**Background:**

Task-oriented rehabilitation supported by exoskeletons has the potential to increase therapy intensity, personalization, and accessibility. However, to achieve fully automatic treatment, robotized systems need to analyze therapy in a more complex way than only based on reference trajectories following.

**Objective:**

This study aimed to investigate the effects of an intelligent, context-aware control algorithm for an upper limb rehabilitation exoskeleton on patients’ musculoskeletal engagement, compared with constant-admittance robot-assisted therapy, and conventional physiotherapist-guided treatment.

**Methods:**

A single-session experimental study was conducted with 34 adult participants performing 6 activities of daily living under 3 therapy modes: robot-assisted therapy with constant admittance, robot-assisted therapy with an intelligent assist-as-needed algorithm, and physiotherapist-guided therapy. Muscle activity was assessed using surface electromyography of 8 upper limb muscle groups, while joint kinematics were recorded using inertial measurement units. Metrics included electromyography power, muscle activation time, joint range of motion, and Burst Duration Similarity Indices. Statistical comparisons were performed using the *t* test and the Mann-Whitney *U* test depending on data normality.

**Results:**

Results indicate that the intelligent control strategy engages the musculoskeletal system at least as effectively as constant-admittance control across all exercises. At the same time, more motion control is given to the patient, which is consistent with the principles of neuroplastic motor learning. Compared with physiotherapist-guided therapy, robot-assisted treatment with intelligent control elicited significantly higher and more consistent muscular engagement. Intelligent assistance also modified joint-level motion patterns by reducing compensatory movements, particularly in shoulder-elbow coupling, while maintaining functional task execution. Muscle activation timing patterns during intelligent robot-assisted therapy were more consistent with robotic control than with manual therapy, reflecting altered movement strategies.

**Conclusions:**

These findings demonstrate that context-aware, intelligent control in rehabilitation exoskeletons can promote active patient participation, reduce compensatory behaviors, and maintain physiologically meaningful muscle engagement. The proposed approach exceeds the results of recent similar studies, being a promising step toward effective, minimally supervised, and task-oriented rehabilitation.

## Introduction

### Background

Upper extremity impairment is among the most common consequences of neurological conditions, including stroke. Moreover, it is severely affecting functional independence in activities of daily living (ADLs) and requires intensive, long-term physiotherapy, which significantly burdens the health care systems [1]. Despite the recognized clinical importance of rehabilitation, the global supply of trained physiotherapy professionals remains critically insufficient relative to demand, even 10-fold in less developed European countries [2]. This structural deficit is even more serious when combined with geographic inequalities in the reachability of specialist centers and place-related qualified personnel ready to perform physically demanding work [3]. Therefore, this paper is focused on the proposition of a technological approach to robot-aided task-oriented treatment, mitigating the problem of medical staff shortages.

### Robot-Aided Task-Oriented Treatment

Task-oriented rehabilitation constitutes the foundation of contemporary physiotherapy, and its primary objective is to restore patients’ ability to perform ADLs with the highest possible level of independence. From the physiotherapist’s perspective, the key factors are not merely the repetitiveness of movement but, above all, its functional nature, appropriate engagement of the musculoskeletal system, and the continuous adjustment of the level of assistance to the patient’s current capabilities [4].

The literature indicates that robot-assisted rehabilitation may improve motor function and quality of life in patients with neurological impairments to a degree comparable with that achieved with conventional therapies, particularly with respect to movement repetitiveness, training intensity, and the facilitation of neuroplasticity [5]. Beyond the aspects of repetitiveness and functionality, current technology enables a key element of therapy—personalization. Tailoring the therapy to the individual patient may significantly enhance its effectiveness [6].

Moreover, automation enhances physiotherapists’ capabilities as telemedicine becomes a clinical standard in many countries [7]. This is particularly important due to the shortages in medical staff—robotization of physical therapy can bring a significant benefit of early treatment at high doses to patients. Nevertheless, this requires safe operation, which is guaranteed by continuously adjusting the device-human interaction based on the analyzed operation. Apart from the risk of physical damage, potential discomfort and unexpected situations during treatment can decrease trust in the robot. This can become a significant obstacle in encouraging people to use robotized treatment instead of human-conducted treatment—even despite the presented benefits [8].

In these aspects, solutions that enable dynamic, context-aware adjustment of exoskeleton assistance become particularly important, analogous to the decisions a physiotherapist makes during manually guided therapy. The integration of the assessment of novel task-oriented exercises with intelligent control algorithms may represent a key factor in enhancing the effectiveness of robot-assisted rehabilitation by promoting more effective engagement of the musculoskeletal system and supporting the patient’s active participation in the rehabilitation process [6].

### Control Strategies

Contemporary rehabilitation robots, for example, end-effectors or wearable exoskeletons, seek to deliver repeatable therapy while controlling interaction forces and preserving active patient participation [9]. A prevalent control architecture consists of a fast inner loop enforcing joint- or task-space motion and an outer impedance or admittance loop shaping the dynamics through a virtual mass–spring–damper driven by measured interaction forces, balancing tracking performance and contact sensation [10,11].

A key challenge is that admittance stability and transparency are determined by the complete closed-loop chain rather than by the virtual dynamics alone. Stability margins depend strongly on force sensing and filtering, computation or communication delays, and the bandwidth of the inner motion loop [12]. Excessive force filtering introduces phase lag and can destabilize human-robot interaction, especially when rendering low virtual inertia to encourage the patient’s motion. Practical guidelines, therefore, emphasize minimizing force filtering, injecting sufficient virtual damping, and explicitly preserving passivity of the coupled system [12,13]. Clinically, this matters because excessive mechanical resistance reduces the patient’s voluntary contribution to movement, significant for neurorehabilitation.

Within this framework, recent developments focus on adapting assistance to the specific requirements of the rehabilitation task. Rather than using fixed interaction dynamics, controllers are increasingly designed to adjust the assistance based on inferred user intent and on task-dependent objectives. Yu et al [14] proposed a proportional-integral-derivative controller–type admittance and highlighted implementation aspects, including gravity compensation and sensor fusion for reference generation. At the same time, task-space formulations have been shown to reduce unnecessary energy exchange during exercises [15]. Beyond time-domain tuning, admittance shaping may specify target natural frequency, damping, and low-frequency gain for the human-robot pair [16]. Also, variable admittance strategies adapt parameters online based on interaction force, tracking error, or collaboration state; for example, a collaboration observer can detect unsafe interaction and temporarily raise virtual inertia and damping to recover stability [17]; a task-oriented variable admittance can modulate assistance across different exercise phases [18]. Assist-as-needed (AAN) formulations embedded in variable admittance controllers are emerging [19], with analogous concepts explored in lower limb devices, for example, strength-index–based AAN for a hip exoskeleton [20]. From a rehabilitation perspective, AAN strategies are motivated by the principle that the optimal therapy should be focused on providing just enough assistance to enable successful task completion while remaining the other part to the patient’s motor system.

Various metrics, in addition to standard robotics indices such as tracking errors, are used to assess the efficacy of these control approaches; for example, the Fugl-Meyer assessment, electromyography (EMG), or electroencephalography [21]. Results from such trials yield important conclusions, as shown in Aurich-Schuler et al [22], where rehabilitation with different control settings of the Lokomat showed that more permissive modes can increase muscle activation and promote more physiological patterns. Crucially, this finding highlights that control-level choices, not just exercise selection, directly determine the biomechanical profile of the therapy session.

The rule-based admittance adaptation can be further personalized. Learning-augmented admittance uses recurrent neural networks to fine-tune virtual inertia and damping for improved tracking [23]. Others incorporate physiological metrics, such as muscular manipulability, to direct assistance to where the patient’s capability is low [24]. An important venue is dynamic compensation. Because rehabilitation motions are often slow, many systems emphasize gravity compensation rather than full inertial dynamics, using various learning-based methods [25,26].

A critical question is then whether an algorithm that autonomously adapts support based on contextual assessment of task performance, rather than manual parameter tuning, can produce measurably different musculoskeletal engagement compared with both fixed-admittance robotic control and conventional physiotherapy.

### Aim of the Study

Taken together, these results motivate context-aware tuning of assistance settings and evaluation of whether such settings measurably improve the rehabilitation process. Despite the advances, selecting and updating controller parameters are still most often manual, task-specific processes, and controllers that perform well in one exercise may not generalize when the task context or patient capability changes. Moreover, improvements in stability or tracking do not necessarily translate into better rehabilitation outcomes, which even strengthens the case for algorithms that assist based on contextual assessment and for outcome-oriented indices.

Prior research conducted by the authors showed that using an intelligent algorithm to set support for exoskeleton-aided therapy, based on a contextual assessment of new exercises rather than constant support settings, results in more accurate exercise performance, although more control is given to the patient. This shows that providing more support upon detecting significant errors and less support during the rest of the operation increases motion quality. Moreover, it remains in line with the principles of neuroplastic exercising, as the patient is more independent while deciding on motion patterns. Despite confirmation of superiority in terms of patient participation in therapy and reduced need for constant support, the impact on the patient’s musculoskeletal system must be analyzed.

This study is a natural continuation of the investigation process, focusing on the biomechanics of patients. It aims to examine whether the developed algorithms engage patients at a higher level than conventional control algorithms and physiotherapist-conducted therapy. This is determined based on statistical tests performed using metrics of muscular activity and joint range of motion (ROM). The test hypotheses aim to assess whether EMG- and joint motion (JM)–related parameters remain higher for the intelligent mode than for the control of the same robot with constant-admittance active therapy than the manual physiotherapy. The precise formulation of these is presented in the Metrics subsection of the Methods section.

The results of the experiment will enable an evidence-based comparison of the developed approach to providing therapy with exoskeletons with alternative, well-established approaches. This is particularly significant, as the proposed therapy methodology also enables easy treatment scaling and personalization and, as a consequence, brings robot-aided therapy closer to a homelike environment with minimal specialist supervision (also remote).

## Methods

### Ethical Considerations

The experiments were carried out under the KB/132/2024 approval of the Bioethical Committee of the Medical University of Warsaw. Written informed consent was obtained from all of the participants involved in this study. It involved a group of 34 volunteers (with no compensation) who had given written informed consent to participate. All of the personal data were anonymized as described within the participant information card. The group was heterogeneous in terms of age (23‐65 years, 
*μ*=36.2), sex, and medical conditions (cardiological, neurological, and orthopedical). An a priori power analysis conducted in G*Power software (Heinrich-Heine-Universität Düsseldorf) indicated that, assuming a within-subject effect size of Cohen *d*=0.4, α=.05, power=0.95, 2-group tests used within this paper, and 2-tailed analysis, 34 participants with 5 repetitions were required [27].

### Experimental Procedure

The experimental phase consisted of performing a standardized set of 6 exercises with 5 repetitions each, all in 3 modes dependent on the support of the therapy, also with the rehabilitation exoskeleton. Every exercise reference motion was recorded by the device during physiotherapist-guided exercises, uniquely for each patient during a physiotherapist-guided familiarization session prior to the experimental protocol. Thanks to this, individual limitations and natural motion patterns were taken into account. The modes used in the study included the following:

1. Mode 1: active therapy with the support of the SmartEx-Home rehabilitation exoskeleton controlled with the admittance algorithm. The device adapted to the intended motion of a patient based on measurements from multiaxial force sensors integrated with the exoskeleton and corrected the motion proportionally to the trajectory deviation. The admittance is constantly set to 0.5.
2. Mode 2: active therapy with the support of the SmartEx-Home rehabilitation exoskeleton controlled with the intelligent algorithm. The algorithm assesses reference trajectories (not preprogrammed) for potential functional errors and anatomical compensations and assists the patient as needed to minimize the risk of their occurrence. This enabled the patient to modify the motions as within mode 1, but the corrections were connected not only to the trajectory deviations but also to the contextually analyzed errors. The precise operation of this mode is described in the complementary paper (Falkowski P and colleagues, unpublished data, 2026).
3. Mode 3: active therapy with the support of the physiotherapist and correction of motions as within real-life treatment.

**Figure 1.**
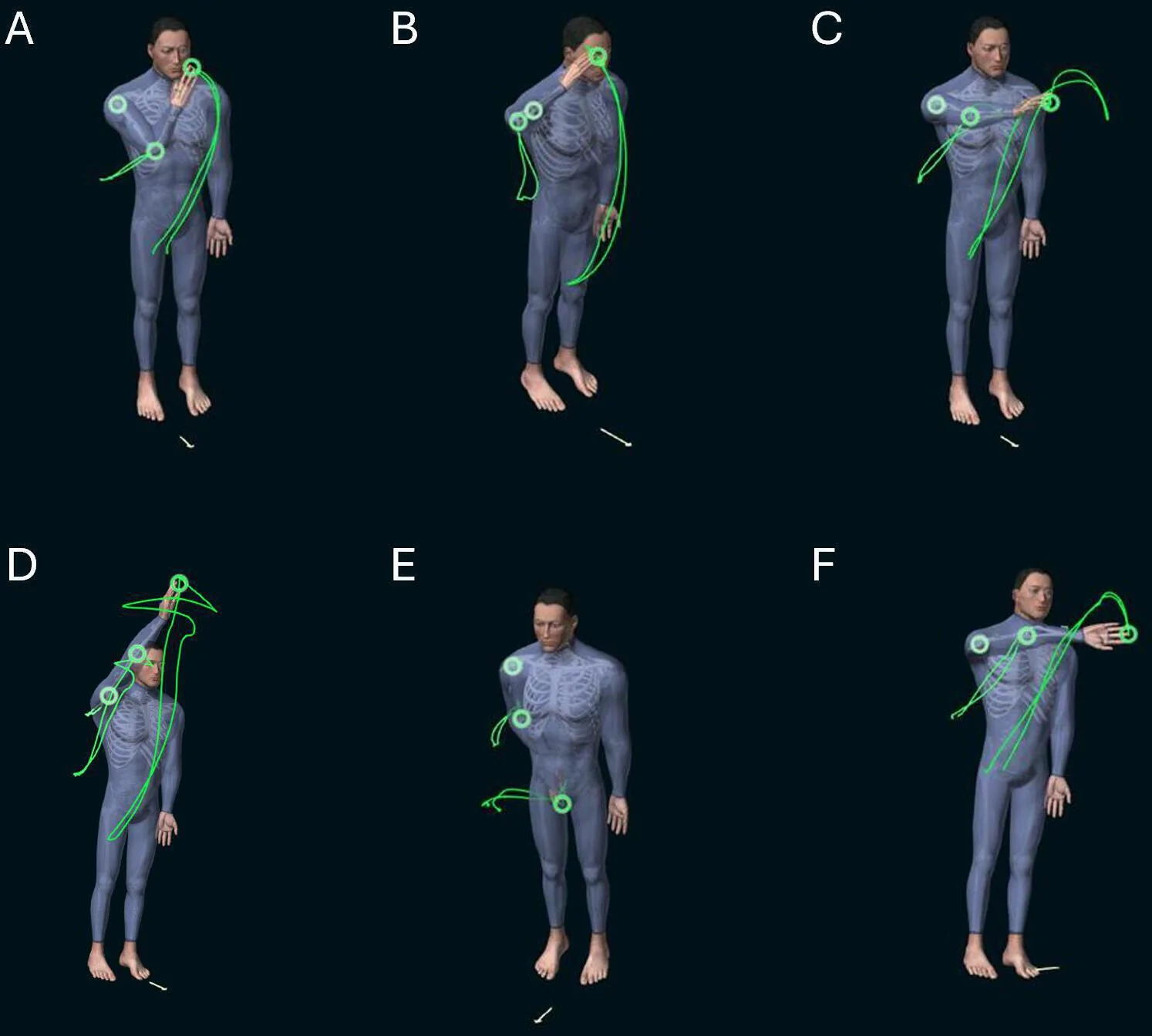
Visual presentation of exercise trajectories (top: A, B, and C; bottom: D, E, and F).

The exercises used in the procedure are visualized in Figure 1 and include (A) exercise 1: snacking, (B) exercise 2: answering the phone call, (C) exercise 3: carrying the shopping bags, (D) exercise 4: shutting the curtains, (E) exercise 5: wiping buttocks, and (F) exercise 6: pouring a drink.

### SmartEx-Home Exoskeleton

**Figure 2.**
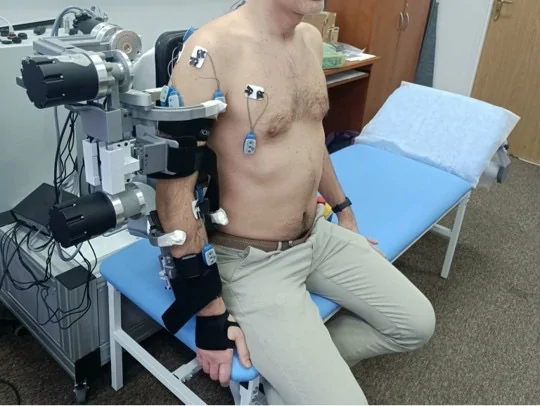
Experimental setup.

The SmartEx-Home exoskeleton for the upper extremity on a mobile platform was used in the study [28,29]. It has 5 degrees of freedom—3 driven (shoulder abduction or adduction, shoulder flexion or extension, and elbow flexion or extension) and 2 passive—open sliding bearings [30] (shoulder and radioulnar rotations). The wrist motion remains free. A patient is connected to the robot’s construction at the torso, arm, and forearm with elastic straps, with the 2 extremity attachments equipped with 3-axis force sensors. The exoskeleton can provide passive or active treatment, also in AAN mode. The last one involves an intelligent algorithm, which can assess any exercise reflecting the ADL contextually. This includes finding similarities between new exercises and preprogrammed ADLs [31]. Then it assesses trajectory following, anatomical compensations [32], and functional errors. Assistance is set based on the patients’ performance. A patient performing an experimental routine with the exoskeleton is presented in Figure 2. While exercising, patients can see a visual of a human model on screen—1 representing their current position, and 1 representing the desired position.

### Measuring

The study aimed to assess the impact on the patients’ musculoskeletal system. Therefore, metrics related to muscle activity and the involved ranges of motion were selected. To monitor them, the Noraxon Ultium system with inertial moment units (IMU sensors) and EMG sensors was used to measure the process. Additionally, 2 integrated cameras were recording exercises to facilitate the possibility of validating analyses and dividing datasets into repetitions.

Four IMU sensors were attached to the following regions of every patient’s body according to the system’s documentation: (1) upper thoracic: below C7 in line with the spinal column but high enough to not be affected by upper trapezius muscle movement; (2) upper arm: midway between the shoulder and elbow joints, lateral to the bone axis; (3) forearm: posterior and distal, where there is a low amount of muscle tissue; and (4) hand: dorsal.

The calibration process was performed according to the system’s documentation. The calibration position was static—standing or seated for patients with severe disabilities of the lower extremities. This involves setting all joints to the zero-degree position (or 90 degrees for those with disabilities) and activating the calibration mode. The standing calibration position requires all joints to be in the neutral, zero position, with the palms aligned with the sagittal plane. The seated calibration position requires a patient to be seated with the elbow, hip, and knee at 90-degree flexion.

As a result, the following 5 JMs were computed as time series in degrees regarding base position: shoulder abduction or adduction (JM 1), shoulder flexion or extension (JM 2), shoulder rotations (JM 3), elbow flexion or extension (JM 4), and wrist radial rotations (JM 5). The computation was performed automatically in MR 4.2 software (Noraxon) based on the registered signals for IMU sensors. However, each time series also underwent an unwrapping signal process to eliminate potential discontinuities resulting from the assumed ranges of motion while capturing the original sensors’ Euler angles [33].

Eight EMG sensors were attached with surface gel electrodes according to *Noraxon* [34] and *SENIAM* [35] guidelines on the following muscles: middle deltoid (EMG 1), posterior deltoid (EMG 2), pectoralis major (EMG 3), teres major (EMG 4), latissimus dorsi (EMG 5), biceps brachii (EMG 6), triceps brachii (medial head; EMG 7), and brachioradialis (EMG 8). EMG signals were collected at 2000 Hz using Noraxon’s Ultium EMG system and processed offline following the manufacturer’s guidelines and according to analog experimental practice [36,37]. The processing chain included the following steps: (1) high-pass filter: applied a 10-Hz IIR Butterworth filter to remove motion artifacts and baseline drift (standard setting for chosen tasks that are not very dynamic when performed with a robot), (2) low-pass filter: applied a 500-Hz IIR Butterworth filter to keep relevant EMG content and remove high-frequency noise, (3) rectification: converted the bipolar signal to positive values only using full-wave rectification, (4) smoothing: used a 200-ms moving average to create a smooth envelope (recommended for our type of motion recordings), and (5) normalization: divided by the functional muscular voluntary contraction level [38] from an automatically chosen maximum value over a 1000-ms window. The gathered data were then statistically analyzed with distinction between modes, exercises, and channels (different for EMG and IMU). This enabled drawing global conclusions and considering the significant muscle activity and JM during various exercises.

### Metrics

The theses from the introduction were analyzed using four metrics [39]:

- *maxEMG*: measure reflecting maximum activity generated with the muscle within the exercise, computed as the maximum of the EMG signal.
- *iEMG*: measure reflecting power (midpoint integral) of the muscular activity, computed according to the formula (1), where *EMG* is the value of EMG signal, and *t* is time.

(1) $$iEMG=\frac{1}{2}\sum_{n=2}^{N}\left(EMG\left(n\right)+EMG\left(n-1\right)\right)\cdot \left(t\left(n\right)-t\left(n-1\right)\right)$$

- *T_act_*: measure reflecting the time within the exercise when the muscle is active, computed according to the formula (2), where N is the number of samples in the signal, and *act*(*n*) is the activation function calculated according to the formula (3) with the levels set based on literature sources [40].

(2) $$T_{act}=\sum_{n=2}^{N}act\left(n\right)\cdot \left(t\left(n\right)-t\left(n-1\right)\right)$$

(3) $$act\left(n\right)=\{\begin{array}{c} \begin{array}{ccc} 0 & if & EMG\left(n\right)§amp;lt;15\%∙fMVC \end{array}\\ \begin{array}{ccc} 1 & if & EMG(n)\geq 15\%∙fMVC \end{array} \end{array}$$

- ROM: measure reflecting the range of a JM used within the exercise, computed as the minimal value of the joint position subtracted from its maximum value.

For statistical analysis, the metrics were grouped by combinations of modes, exercises, and channels (8 muscular groups for EMG-based metrics and 5 JMs for ROM). These were treated as separate population groups. First of all, each of these was validated for normality using the Shapiro-Wilk test. As the groups were relatively large (all >150 samples), the 1-tailed *t* test was used for those that were normally distributed. For the nonnormal distributions, the Mann-Whitney *U* test was used [41]. Both test types were conducted using MATLAB (MethWorks) functions that enable validation of 1-sided alternative hypotheses.

In fact, 2 subsequent statistical tests were conducted for each dataset. To prove the relation with the constant-admittance therapy, the first hypothesis (null and alternative) was formed:

- Hypothesis 0: Effectiveness metrics for the intelligent algorithm are statistically *comparable to or lower* than the ones for the nonintelligent algorithm.
- Hypothesis 1: Effectiveness metrics for the intelligent algorithm are statistically *higher* than those for the nonintelligent algorithm.

Then the second hypothesis (null and alternative) was formed:

- Hypothesis 0: Effectiveness metrics for the intelligent algorithm are statistically *comparable to or greater* than the ones for the nonintelligent algorithm.
- Hypothesis 1: Effectiveness metrics for the intelligent algorithm are statistically *lower* than those for the nonintelligent algorithm.

If hypothesis 0 is rejected during the first test, it is assumed that the compared metrics are greater than the reference metrics (further marked G). For this statistical test result, the *P* value from the first test is presented. If it fails to reject hypothesis 0 in the first test and hypothesis 0 is rejected in the second test, it is assumed that the compared metrics are lower than the reference metrics (further marked L). If it fails to reject hypothesis 0 in both tests, it is assumed that the compared metrics are statistically equal to the reference metrics (meaning that no significant difference is observed; further marked E). For these 2 statistical test results, the *P*-value from the second test is presented.

Analogous hypotheses were formed for comparison with the conventional physiotherapy, manually guided by a physiotherapist. To validate the hypothesis, all the presented metrics beforehand reflecting muscular activity and joint mobility were used. No family-wise error correction was applied; results should be interpreted in an exploratory context, particularly for comparisons. However, additional ANOVA comparison was performed for the whole groups of results for different modes, not dividing them based on EMG channels, JMs, or exercises.

Additionally, BDSI (Burst Duration Similarity Index) values were computed for comparison of activity of muscles during mode 2 with mode 1 and mode 2 with mode 3 according to the formula (4), including activity functions computed for 2 compared muscular groups according to formula (3) [39]. Before, the time series for the compared EMG signals were approximated and normalized to have the same number of elements, as the comparison is connected to the phase of the realized motion, and their durations varied.


(4) $$BDSI=\frac{1}{N}\sum_{n=1}^{N}XNOR\left(act_{1}\left(n\right),act_{2}\left(n\right)\right)$$

BDSI indices were then grouped by muscle group and exercise (without any other distinguishing factors). The mean and standard deviation were then computed for them. This enabled analysis of differences in the moments of muscle activation across motions and between therapy modes.

## Results

### Overview

The results in this section are presented with the distinction between muscular activity, ROM, and motion patterns comparisons. They are analyzed for exercises and either EMG channels or single JMs combined with exercises separately to draw the precise initial conclusions on the impact of the intelligent mode on performing treatment. However, to assess it at the global scale, the comparison for all the classes combined was additionally performed.

### Muscular Activity

Each exercise represents a different ADL. Therefore, the motions differ significantly, as shown by the activity of different muscular groups and the range of JM. Based on the results from mode 3, the most critical items for each exercise were identified. Analysis for muscular groups was performed based on mean values and standard deviations for EMG values and the time of activation during exercises. Analysis for JMs was performed considering the mean ranges of motions used in the exercise, compared with the ranges of motion used for all of the exercises.

The first conclusion from this analysis was that the latissimus dorsi was contracted at a stable level close to the functional muscular voluntary contraction during most exercises to stabilize the upper body of patients. Also, pectoralis major and teres major were relatively active during the exercises, with the most significant activity during answering the phone and carrying the shopping bags for ADLs, respectively. Posterior deltoid, the muscle acting antagonistically to it, was continuously active at a significantly lower level. Similarly, biceps brachii and brachioradialis were active for long periods but with stronger contractions. The latter was also outstandingly active during answering the phone call exercise, due to the rotation of the hand close to the ear. The biceps brachii were relatively inactive during exercises, except for the wiping-the-buttocks movement.

In terms of joint kinematics, patients used mainly abduction or adduction and flexion or extension of the shoulder, along with flexion or extension of the elbow. A relatively low amount of shoulder flexion or extension was observed only for wiping buttocks, while low elbow flexion or extension was observed for pouring a drink during ADL. Shoulder rotations were activated mainly during exercises 2, 3, and 4, while wrist radial rotation was used mainly for exercises 5 and 6.

In terms of muscular activity, 2 different metrics were used to compare modes of exercise. *iEMG* was used to represent the power exerted by every monitored muscular group during exercises. Comparison of intelligent control in the constant-admittance mode shows that, for most combinations, the results are statistically comparable, although the metric means during intelligent treatment are typically higher (Table 1). In a few cases for EMG 2, and EMG 6-8 muscular groups, the results are statistically better, while for EMG 1, the results are lower in exercise 4. *T_act_* was used as a measure reflecting the time of the muscles’ activity. For compared modes 2 and 1, the times are statistically equal for most of the exercises and muscular groups and are higher during intelligently controlled treatment for 5 combinations of those (Table 2). This metric was not greater for the constant-admittance mode for any of the considered cases.

The outcomes prove that the robot controlled with intelligent algorithms examined in this study tends to engage the muscular system of a patient at least at the same level as the one controlled with the constant-admittance mode. At the same time, previous research evidenced that the former gives more control over the motion to the patient, which is applicable for neuroplastic rehabilitation [42]. Moreover, using the former results in more accurate following of the recorded trajectories. The results from the presented analysis confirm that the newly developed method is at least as effective as the well-established approach to active therapy with rehabilitation robots [2].

Moreover, the *iEMG* and *T_act_* parameters were compared between modes 2 and 1 for the whole gathered dataset, without distinction on EMG channels and exercises. The results of statistical analysis rejected the hypothesis that the effectiveness metrics for the intelligent algorithm are statistically comparable with or lower than the ones for the nonintelligent algorithm for both parameters (μ=105.42, μ₀=99.57, *P*<.001, and μ=3.39, μ₀=3.14, *P*<.001, respectively, for *iEMG* and *T*_*act*_).

Analogical analyses were performed to compare mode 2 with physiotherapist-provided treatment. The results are significantly better for the therapy provided by the robot (Tables 3 and 4). While validating the outcomes, it was observed that exercises provided by a physiotherapist without the device were significantly faster during short sessions than those designed with the exoskeleton. Nevertheless, this naturally affected not only 𝑇_𝑎𝑐_*_t_* parameters but also *iEMG*, which, as reflecting muscle work, should not be time-dependent. As observed, patients feel more responsible for the motions performed while exercising with the exoskeleton. Hence, they engage more physically and cognitively in the treatment.

Also, the *iEMG* and 𝑇_𝑎𝑐_*_t_* parameters were compared between modes 2 and 3 for the whole gathered dataset, without distinction on EMG channels and exercises. The results of statistical analysis rejected the hypothesis that the effectiveness metrics for the intelligent algorithm are statistically comparable with or lower than the ones for the nonintelligent algorithm for both parameters (μ=105.42, μ₀=35.27, *P*<.001, and μ=3.39, μ₀=1.08, *P*<.001, respectively, for *iEMG* and *T_act_*).

Moreover, physiotherapists tend to become physically tired during their work [3]. Even if they focus on faster motions of patients, they tend to take longer breaks between them. Long-term results regarding the time to muscle activation are expected to be significantly lower than those observed during robot-aided treatment with the proposed rehabilitation exoskeleton.

**Table 1.** Statistical results for mode 2 versus mode 1; metric: *iEMG*^a^^,^^b^.

	Exercise 1	Exercise 2	Exercise 3	Exercise 4	Exercise 5	Exercise 6
EMG^c^ 1	E (*P*=.91, μ=108.35, μ₀=101.14)	E (*P*=.13, μ=94.66, μ₀=102.86)	E (*P*=.90, μ=81.19, μ₀=82.03)	L (*P*=.04, μ=162.51, μ₀=178.58)	E (*P*=.49, μ=43.54, μ₀=37.62)	E (*P*=.21, μ=98.71, μ₀=95.00)
EMG 2	G (*P*=.01, μ=96.60, μ₀=78.51)	E (*P*=.94, μ=81.20, μ₀=69.86)	G (*P*=.005, μ=85.67, μ₀=68.36)	E (*P*=.58, μ=117.61, μ₀=105.54)	E (*P*=.36, μ=83.28, μ₀=80.21)	E (*P*=.93, μ=91.02, μ₀=77.47)
EMG 3	E (*P*=.93, μ=154.21, μ₀=140.95)	E (*P*=.83, μ=141.66, μ₀=130.08)	E (*P*=.75, μ=133.83, μ₀=128.89)	E (*P*=.86, μ=181.94, μ₀=167.09)	E (*P*=.94, μ=114.52, μ₀=100.90)	E (*P*=.81, μ=153.98, μ₀=145.06)
EMG 4	E (*P*=.61, μ=141.42, μ₀=139.53)	E (*P*=.49, μ=128.04, μ₀=128.99)	E (*P*=.41, μ=114.66, μ₀=117.88)	E (*P*=.45, μ=172.11, μ₀=173.52)	E (*P*=.93, μ=112.52, μ₀=96.47)	E (*P*=.67, μ=129.44, μ₀=123.30)
EMG 5	E (*P*=.76, μ=134.83, μ₀=128.56)	E (*P*=.31, μ=120.47, μ₀=127.44)	E (*P*=.63, μ=111.12, μ₀=113.91)	E (*P*=.59, μ=157.19, μ₀=156.54)	E (*P*=.65, μ=103.69, μ₀=103.41)	E (*P*=.72, μ=125.55, μ₀=120.19)
EMG 6	E (*P*=.94, μ=91.72, μ₀=81.08)	G (*P*=.03, μ=95.96, μ₀=78.16)	E (*P*=.62, μ=85.03, μ₀=82.51)	E (*P*=.55, μ=107.30, μ₀=103.72)	E (*P*=.82, μ=69.98, μ₀=62.28)	E (*P*=.59, μ=86.06, μ₀=86.54)
EMG 7	G (*P*=.05, μ=78.80, μ₀=71.89)	E (*P*=.71, μ=67.00, μ₀=66.45)	E (*P*=.80, μ=62.31, μ₀=62.84)	E (*P*=.78, μ=92.30, μ₀=84.77)	E (*P*=.59, μ=60.38, μ₀=57.02)	E (*P*=.83, μ=63.38, μ₀=58.93)
EMG 8	E (*P*=.78, μ=93.34, μ₀=82.14)	E (*P*=.25, μ=81.55, μ₀=88.39)	G (*P*=.03, μ=83.20, μ₀=72.14)	E (*P*=.88, μ=95.61, μ₀=88.24)	E (*P*=.93, μ=73.04, μ₀=56.43)	E (*P*=.90, μ=86.01, μ₀=73.02)

a *iEMG:* integrated EMG.

b E: statistically equal for both modes; L: statistically lower for mode 2 compared with mode 1; G: statistically greater for mode 2 compared with mode 1.

c EMG: electromyography.

**Table 2.** Statistical results for mode 2 versus mode 1; metric: *T*_*act*__*^.^*_^a^.

	Exercise 1	Exercise 2	Exercise 3	Exercise 4	Exercise 5	Exercise 6
EMG^b^ 1	G (*P*=.03, μ=3.50, μ₀=3.06)	E (*P*=.34, μ=3.19, μ₀=3.27)	G (*P*=.009, μ=2.96, μ₀=2.57)	E (*P*=.34, μ=4.52, μ₀=4.68)	E (*P*=.84, μ=1.12, μ₀=0.98)	E (*P*=.38, μ=2.81, μ₀=2.88)
EMG 2	G (*P*=.02, μ=3.06, μ₀=2.52)	E (*P*=.95, μ=2.56, μ₀=2.14)	G (*P*=.003, μ=2.79, μ₀=2.14)	E (*P*=.62, μ=3.77, μ₀=3.66)	E (*P*=.37, μ=2.58, μ₀=2.42)	E (*P*=.93, μ=2.94, μ₀=2.32)
EMG 3	E (*P*=.95, μ=4.74, μ₀=4.19)	E (*P*=.69, μ=4.27, μ₀=4.16)	E (*P*=.87, μ=4.15, μ₀=3.80)	E (*P*=.91, μ=5.43, μ₀=4.94)	E (*P*=.92, μ=3.52, μ₀=3.07)	E (*P*=.74, μ=4.64, μ₀=4.32)
EMG 4	E (*P*=.91, μ=4.57, μ₀=4.31)	E (*P*=.81, μ=4.36, μ₀=4.21)	E (*P*=.77, μ=4.08, μ₀=3.97)	E (*P*=.788, μ=5.30, μ₀=5.00)	E (*P*=.83, μ=3.88, μ₀=3.62)	E (*P*=.63, μ=4.54, μ₀=4.42)
EMG 5	E (*P*=.58, μ=5.07, μ₀=4.81)	E (*P*=.76, μ=4.81, μ₀=4.55)	E (*P*=.77, μ=4.58, μ₀=4.36)	E (*P*=.59, μ=5.60, μ₀=5.46)	E (*P*=.62, μ=4.09, μ₀=3.95)	E (*P*=.64, μ=4.76, μ₀=4.69)
EMG 6	E (*P*=.77, μ=2.51, μ₀=2.40)	G (*P*=.02, μ=2.40, μ₀=1.98)	E (*P*=.69, μ=2.16, μ₀=2.07)	E (*P*=.90, μ=2.91, μ₀=2.63)	E (*P*=.66, μ=1.62, μ₀=1.62)	E (*P*=.59, μ=2.32, μ₀=2.32)
EMG 7	G (*P*=.03, μ=2.34, μ₀=2.05)	E (*P*=.95, μ=2.32, μ₀=2.01)	E (*P*=.81, μ=2.11, μ₀=2.01)	E (*P*=.81, μ=3.20, μ₀=2.84)	E (*P*=.74, μ=1.88, μ₀=1.62)	E (*P*=.60, μ=1.94, μ₀=1.75)
EMG 8	E (*P*=.86, μ=3.05, μ₀=2.60)	E (*P*=.42, μ=2.59, μ₀=2.56)	E (*P*=.92, μ=2.60, μ₀=2.33)	E (*P*=.71, μ=3.05, μ₀=2.93)	E (*P*=.79, μ=2.14, μ₀=1.86)	E (*P*=.79, μ=2.78, μ₀=2.54)

a E: statistically equal for both modes; L: statistically lower for mode 2 compared with mode 1; G: statistically greater for mode 2 compared with mode 1.

b EMG: electromyography.

**Table 3.** Statistical results for mode 2 versus mode 3; metric: *iEMG^a^^,^*^b^*.*

	Exercise 1	Exercise 2	Exercise 3	Exercise 4	Exercise 5	Exercise 6
EMG^c^ 1	G (*P*<.001, μ=108.35, μ₀=26.00)	G (*P*<.001, μ=94.66, μ₀=32.60)	G (*P*<.001, μ=81.19, μ₀=38.97)	G (*P*<.001, μ=162.51, μ₀=79.37)	E (*P*=.37, μ=43.54, μ₀=33.97)	G (*P*<.001, μ=98.71, μ₀=39.68)
EMG 2	G (*P*<.001, μ=96.60, μ₀=14.22)	G (*P*<.001, μ=81.20, μ₀=14.28)	G (*P*<.001, μ=85.67, μ₀=20.39)	G (*P*<.001, μ=117.61, μ₀=39.19)	E (*P*=.64, μ=83.28, μ₀=71.33)	G (*P*<.001, μ=91.02, μ₀=21.88)
EMG 3	G (*P*<.001, μ=154.21, μ₀=36.18)	G (*P*<.001, μ=141.66, μ₀=39.84)	G (*P*<.001, μ=133.83, μ₀=45.47)	G (*P*<.001, μ=181.94, μ₀=57.27)	G (*P*<.001, μ=114.52, μ₀=35.86)	G (*P*<.001, μ=153.98, μ₀=44.77)
EMG 4	G (*P*<.001, μ=141.42, μ₀=25.08)	G (*P*<.001, μ=128.04, μ₀=34.48)	G (*P*<.001, μ=114.66, μ₀=33.94)	G (*P*<.001, μ=172.11, μ₀=48.10)	G (*P*<.001, μ=112.52, μ₀=37.13)	G (*P*<.001, μ=129.44, μ₀=33.78)
EMG 5	G (*P*<.001, μ=134.83, μ₀=29.27)	G (*P*<.001, μ=120.47, μ₀=32.66)	G (*P*<.001, μ=111.12, μ₀=32.12)	G (*P*<.001, μ=157.19, μ₀=40.41)	G (*P*<.001, μ=103.69, μ₀=33.55)	G (*P*<.001, μ=125.55, μ₀=32.43)
EMG 6	G (*P*<.001, μ=91.72, μ₀=35.05)	G (*P*<.001, μ=95.96, μ₀=39.43)	G (*P*<.001, μ=85.03, μ₀=40.09)	G (*P*<.001, μ=107.30, μ₀=49.40)	G (*P*<.001, μ=69.98, μ₀=30.59)	G (*P*<.001, μ=86.06, μ₀=31.62)
EMG 7	G (*P*<.001, μ=78.80, μ₀=14.16)	G (*P*<.001, μ=67.00, μ₀=18.27)	G (*P*<.001, μ=62.31, μ₀=22.26)	G (*P*<.001, μ=92.30, μ₀=33.76)	G (*P*<.001, μ=60.38, μ₀=38.49)	G (*P*<.001, μ=63.38, μ₀=19.59)
EMG 8	G (*P*<.001, μ=93.34, μ₀=35.17)	G (*P*<.001, μ=81.55, μ₀=46.51)	G (*P*<.001, μ=83.20, μ₀=30.33)	G (*P*<.001, μ=95.61, μ₀=39.59)	G (*P*<.001, μ=73.04, μ₀=36.56)	G (*P*<.001, μ=86.01, μ₀=27.18)

a *iEMG:* integrated EMG.

b E: statistically equal for both modes; L: statistically lower for mode 2 compared with mode 3; G: statistically greater for mode 2 compared with mode 3.

c EMG: electromyography.

**Table 4.** Statistical results for mode 2 versus mode 3; metric: 𝑇_𝑎𝑐_*_t_*^a^.

	Exercise 1	Exercise 2	Exercise 3	Exercise 4	Exercise 5	Exercise 6
EMG^b^ 1	G (*P*<.001, μ=3.50, μ₀=0.88)	G (*P*<.001, μ=3.19, μ₀=1.16)	G (*P*<.001, μ=2.96, μ₀=1.27)	G (*P*<.001, μ=4.52, μ₀=1.68)	L (*P*<.001, μ=1.12, μ₀=1.23)	G (*P*<.001, μ=2.81, μ₀=1.26)
EMG 2	G (*P*<.001, μ=3.06, μ₀=0.39)	G (*P*<.001, μ=2.56, μ₀=0.38)	G (*P*<.001, μ=2.79, μ₀=0.63)	G (*P*<.001, μ=3.77, μ₀=1.19)	E (*P*=.94, μ=2.58, μ₀=1.60)	G (*P*<.001, μ=2.94, μ₀=0.66)
EMG 3	G (*P*<.001, μ=4.74, μ₀=1.14)	G (*P*<.001, μ=4.27, μ₀=1.20)	G (*P*<.001, μ=4.15, μ₀=1.38)	G (*P*<.001, μ=5.43, μ₀=1.51)	G (*P*<.001, μ=3.52, μ₀=1.10)	G (*P*<.001, μ=4.64, μ₀=1.41)
EMG 4	G (*P*<.001, μ=4.57, μ₀=1.02)	G (*P*<.001, μ=4.36, μ₀=1.30)	G (*P*<.001, μ=4.08, μ₀=1.22)	G (*P*<.001, μ=5.30, μ₀=1.41)	G (*P*<.001, μ=3.88, μ₀=1.23)	G (*P*<.001, μ=4.54, μ₀=1.18)
EMG 5	G (*P*<.001, μ=5.07, μ₀=1.10)	G (*P*<.001, μ=4.81, μ₀=1.19)	G (*P*<.001, μ=4.58, μ₀=1.15)	G (*P*<.001, μ=5.60, μ₀=1.34)	G (*P*<.001, μ=4.09, μ₀=1.19)	G (*P*<.001, μ=4.76, μ₀=1.18)
EMG 6	G (*P*<.001, μ=2.51, μ₀=1.01)	G (*P*<.001, μ=2.40, μ₀=1.15)	G (*P*<.001, μ=2.16, μ₀=1.12)	G (*P*<.001, μ=2.91, μ₀=1.31)	G (*P*<.001, μ=1.62, μ₀=0.83)	G (*P*<.001, μ=2.32, μ₀=1.07)
EMG 7	G (*P*<.001, μ=2.34, μ₀=0.38)	G (*P*<.001, μ=2.32, μ₀=0.48)	G (*P*<.001, μ=2.11, μ₀=0.65)	G (*P*<.001, μ=3.20, μ₀=0.94)	E (*P*=.800, μ=1.88, μ₀=1.05)	G (*P*<.001, μ=1.94, μ₀=0.56)
EMG 8	G (*P*<.001, μ=3.05, μ₀=0.96)	G (*P*<.001, μ=2.59, μ₀=1.24)	G (*P*<.001, μ=2.60, μ₀=0.91)	G (*P*<.001, μ=3.05, μ₀=1.15)	G (*P*=.01, μ=2.14, μ₀=1.24)	G (*P*<.001, μ=2.78, μ₀=0.98)

a E: statistically equal for both modes; L: statistically lower for mode 2 compared with mode 3; G: statistically greater for mode 2 compared with mode 3.

b EMG: electromyography.

### Ranges of Motion

Apart from positive outcomes, the potential drawbacks in the approach were also spotted. The main areas of those are the ranges of motion actively used by patients during the treatment. One of the 2 most important targets of the treatment, along with increasing muscular activity, is to increase individuals’ range of mobility. While comparing modes of treatment provided by the robot, it can be assumed that the motion patterns differ in terms of pattern for some exercises (particularly exercises 1, 2, and 6; Table 5). Intelligent algorithms used for control generally result in increased shoulder flexion or extension and radioulnar rotation. At the same time, the range of elbow flexion or extension decreases. Theoretically, for patients undergoing therapy of whole extremities, especially neurological, there is a tendency for major problems with the shoulder joint. Moreover, the problem refers specifically to coupled shoulder-elbow flexion motion and ends in unanatomical compensations [1]. As the intelligent mode acts to prevent undesired compensation, the results presented in Table 5 can be treated as a confirmation of the expected system’s operation.

The case is more complex in terms of comparing the developed rehabilitation system with the therapy provided by a human. Definitely, the motion patterns differ in terms of patterns across all the exercises (Table 6). However, the pattern of changes is not as clear as the one described for mode 2 compared with mode 1. Therefore, no definite conclusion about the preferred mechanism can be drawn. It was observed that some of the motions were limited by the construction of the device. This is not different from the problems of other similar constructions [43].

Moreover, it can be partially explained by the fact that the robot in the intelligent mode always reacts to the user’s intention, while the physiotherapist often operates near the pain limits [44]. The second is aimed at maximizing the effects of treatment by increasing ROM quickly. On the contrary, it cannot be applied to the minimally supervised therapy provided by the device, as the reaction time to pain reflexes may be too long, increasing the risk of soft-tissue damage. Given this, it is necessary to specifically focus on changes in the joint ROMs of patients using the system during further long-term effects investigations. This should enable validating whether the robotized therapy can result in slower progress than human-provided and whether the difference can be mitigated by higher-dose treatment [45].

**Table 5.** Statistical results for mode 2 versus mode 1; metric: ROM^a^^,^^b^.

	Exercise 1	Exercise 2	Exercise 3	Exercise 4	Exercise 5	Exercise 6
JM^c^ 1	L (*P*=.005, μ=43.73, μ₀=49.99)	E (*P*=.13, μ=45.40, μ₀=49.22)	E (*P*=.29, μ=36.57, μ₀=41.47)	E (*P*=.55, μ=44.51, μ₀=45.70)	E (*P*=.78, μ=29.57, μ₀=29.80)	G (*P*=.006, μ=37.35, μ₀=38.10)
JM 2	G (*P*<.001, μ=73.30, μ₀=67.23)	G (*P*<.001, μ=76.04, μ₀=62.91)	G (*P*<.001, μ=58.20, μ₀=48.24)	E (*P*=.67, μ=110.51, μ₀=125.59)	G (*P*<.001, μ=32.71, μ₀=25.80)	G (*P*=.001, μ=65.78, μ₀=55.24)
JM 3	E (*P*=.94, μ=73.01, μ₀=55.31)	E (*P*=.07, μ=52.80, μ₀=57.95)	E (*P*=.80, μ=40.24, μ₀=30.61)	E (*P*=.32, μ=183.74, μ₀=188.28)	E (*P*=.31, μ=13.75, μ₀=13.21)	E (*P*=.13, μ=42.91, μ₀=34.33)
JM 4	L (*P*=.002, μ=67.04, μ₀=75.07)	L (*P*=.02, μ=72.82, μ₀=78.52)	E (*P*=.23, μ=54.02, μ₀=55.25)	E (*P*=.50, μ=52.80, μ₀=52.68)	E (*P*=.23, μ=42.22, μ₀=43.99)	L (*P*<.001, μ=50.52, μ₀=60.50)
JM 5	G (*P*=.02, μ=27.54, μ₀=25.23)	E (*P*=.54, μ=26.73, μ₀=28.28)	G (*P*=.002, μ=27.12, μ₀=23.21)	G (*P*<.001, μ=29.78, μ₀=24.74)	G (*P*<.001, μ=21.22, μ₀=17.26)	E (*P*=.90, μ=24.93, μ₀=23.60)

a ROM: range of motion.

b E: statistically equal for both modes; L: statistically lower for mode 2 compared with mode 1; G: statistically greater for mode 2 compared with mode 1.

c JM: joint motion.

**Table 6.** Statistical results for mode 2 versus mode 3; metric: ROM^a^^,^^b^.

	Exercise 1	Exercise 2	Exercise 3	Exercise 4	Exercise 5	Exercise 6
JM^c^ 1	G (*P*<.001, μ=43.73, μ₀=31.43)	G (*P*<.001, μ=45.40, μ₀=34.29)	L (*P*<.001, μ=36.57, μ₀=42.67)	G (*P*=.001, μ=44.51, μ₀=35.82)	G (*P*<.001, μ=29.57, μ₀=24.45)	L (*P*<.001, μ=37.35, μ₀=47.25)
JM 2	G (*P*<.001, μ=73.30, μ₀=47.81)	G (*P*<.001, μ=76.04, μ₀=60.02)	E (*P*=.53, μ=58.20, μ₀=58.07)	G (*P*<.001, μ=110.51, μ₀=99.38)	L (*P*<.001, μ=32.71, μ₀=52.80)	G (*P*<.001, μ=65.78, μ₀=51.96)
JM 3	G (*P*<.001, μ=73.01, μ₀=35.53)	L (*P*=.002, μ=52.80, μ₀=62.94)	L (*P*<.001, μ=40.24, μ₀=61.96)	L (*P*<.001, μ=183.74, μ₀=211.47)	L (*P*<.001, μ=13.75, μ₀=25.18)	E (*P*=.50, μ=42.91, μ₀=42.55)
JM 4	L (*P*<.001, μ=67.04, μ₀=82.23)	L (*P*<.001, μ=72.82, μ₀=89.31)	E (*P*=.70, μ=54.02, μ₀=51.39)	L (*P*<.001, μ=52.80, μ₀=61.28)	G (*P*<.001, μ=42.22, μ₀=32.10)	G (*P*<.001, μ=50.52, μ₀=25.33)
JM 5	G (*P*<.001, μ=27.54, μ₀=15.16)	G (*P*<.001, μ=26.73, μ₀=18.52)	G (*P*=.001, μ=27.12, μ₀=22.09)	G (*P*<.001, μ=29.78, μ₀=20.87)	E (*P*=.10, μ=21.22, μ₀=22.77)	G (*P*<.001, μ=24.93, μ₀=21.40)

a ROM: range of motion.

b E: statistically equal for both modes; L: statistically lower for mode 2 compared with mode 3; G: statistically greater for mode 2 compared with mode 3.

c JM: joint motion

**Figure 3.**
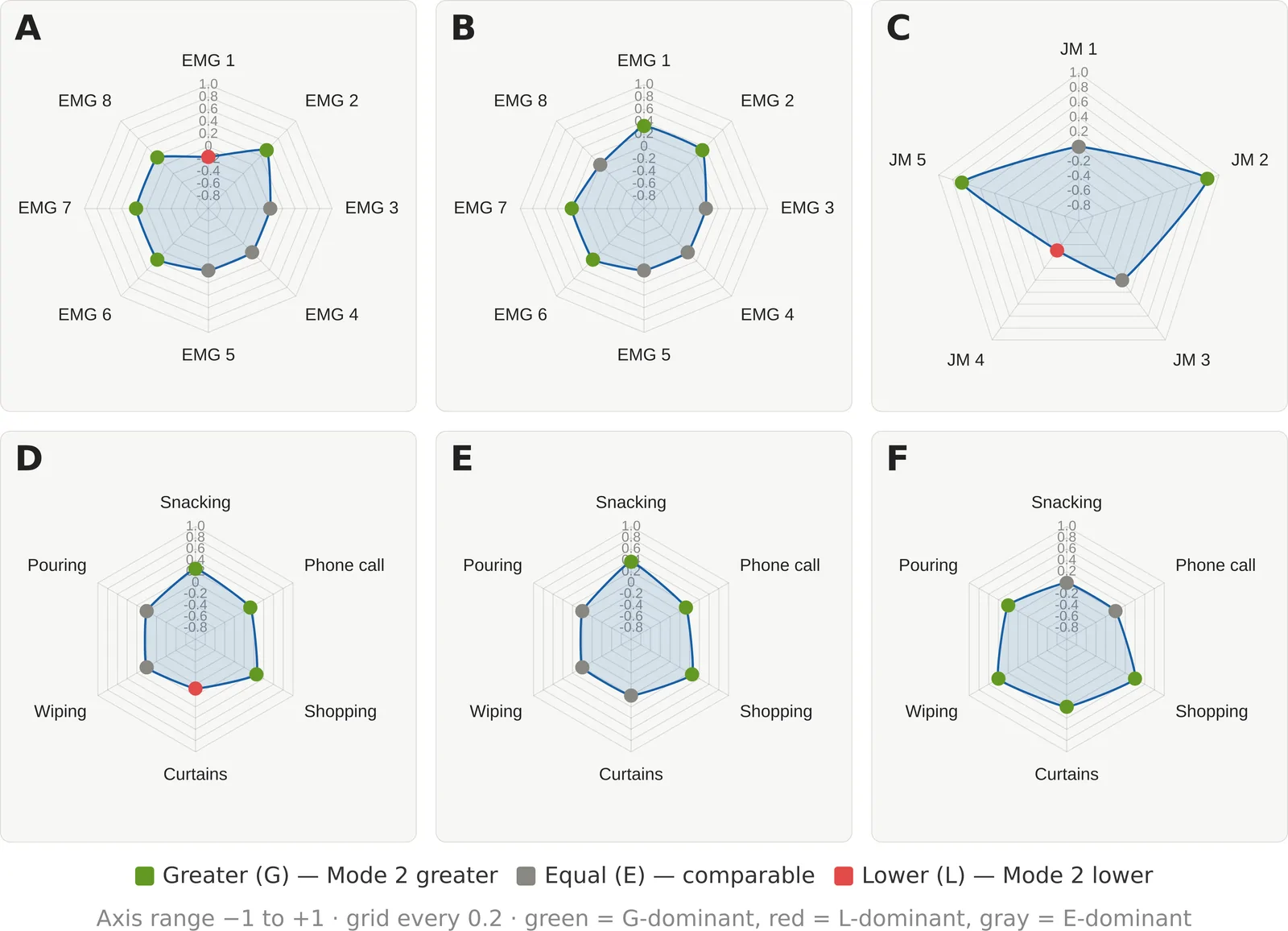
Statistical analysis results for mode 2 versus mode 1 (presented by channel or joint and averaged for all exercises: *iEMG* (A), *T*_*act*_ (B), and ROM (C), presented by exercise and averaged for all channels or joints: *iEMG* (D), *T*_*act*_ (E), and ROM (F); 1 represents results for mode 2 statistically higher for all channels or exercises; −1 represents results for mode 2 statistically lower for all channels or exercises). EMG: electromyography; iEMG: integrated EMG; JM: joint motion; ROM: range of motion; *T*_act_: activation time.

**Figure 4.**
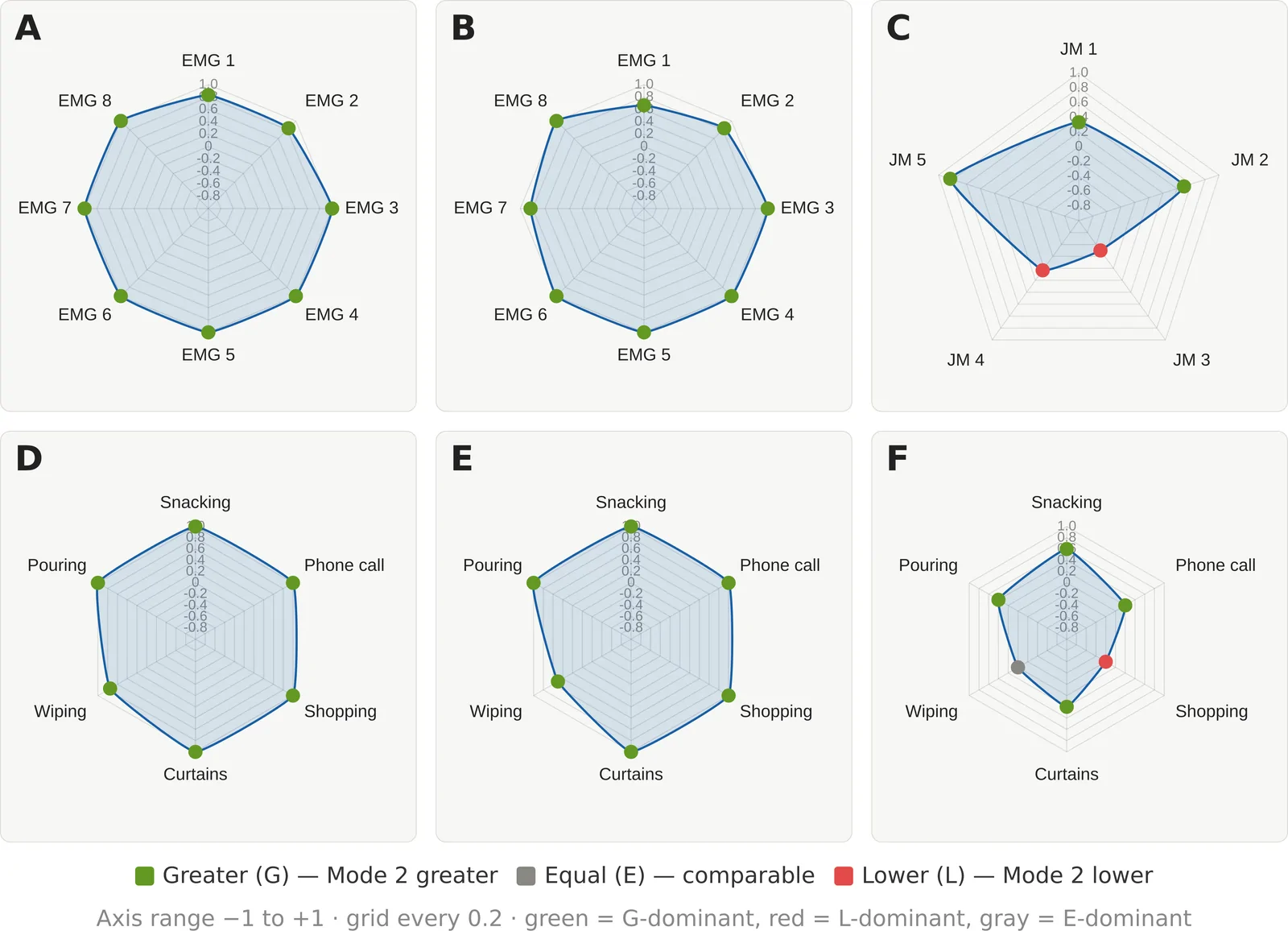
Statistical analysis results for mode 2 versus mode 3 (presented by channel or joint and averaged for all exercises: *iEMG* (A), *T*_*act*_ (B), and ROM (C), presented by exercise and averaged for all channels or joints: *iEMG* (D), *T*_*act*_ (E), and ROM (F); top; 1 represents results for mode 2 statistically higher for all channels or exercises; −1 represents results for mode 2 statistically lower for all channels or exercises). EMG: electromyography; iEMG: integrated EMG; JM: joint motion; ROM: range of motion; *T*_act_: activation time.

Additionally, the ROM was compared between modes 2 and 1 or 3 for the whole gathered dataset, without distinction on JMs and exercises. The result of statistical analysis comparing mode 2 and 3 rejected the hypothesis that this effectiveness metric for the intelligent algorithm is statistically comparable with or lower than the ones for the human-performed trials (μ=47.48, μ₀=46.65; *P*<.001). However, analogical hypothesis was accepted for comparison of modes 2 and 1 (μ=47.48, μ₀=48.13; *P*=.93). Therefore, additional statistical test was performed and simultaneously rejected the hypothesis that this effectiveness metric for the intelligent algorithm is statistically comparable with or higher than the ones for the nonintelligent algorithm (μ=47.48, μ₀=48.13; *P*=.07). Finally, it is assumed that the ROMs engaged within the treatment in both modes 2 and 1 are comparable at the statistical level. The results of EMG- and JMs-related parameters are visualized in Figures 3A-3F and 4A-4F.

### Movement Patterns

Additionally, the BDSI metrics were computed for both comparisons between modes. They reflect how similar muscular activation patterns are when performing the same activity. The similarity of engaging muscles for the robot-assisted treatment modes (Table 7) is higher than for the intelligent mode and exercises supported by a physiotherapist (Table 8). The former are typically in the range of 0.65‐0.8, which is a relatively high result for the different approach [39]. No muscular group nor exercise significantly stands out from the observed pattern.

On the contrary, *BDSI* metrics have significantly more variable values while comparing modes 2 and 3. They range from below 0.5 to 0.8. The first 2 analyzed muscles, the middle and posterior deltoids, differ most in terms of activation when comparing human-provided and intelligent robot-assisted treatment. This is observed across all exercises. Therefore, it is proof that the movements performed during robot-aided treatment differ in terms of mechanisms of muscular control. As this aspect is critical for recalling healthy, functional patterns, further research on long-term effects on patients’ independent functionality should be conducted. The BDSI computed for all the trials resulted in values 0.753 and 0.656 while comparing mode 2 with mode 1 and mode 3, respectively.

**Table 7.** Statistical results for mode 2 versus mode 1; metric: BDSI^a^.

	Exercise 1	Exercise 2	Exercise 3	Exercise 4	Exercise 5	Exercise 6
EMG^b^ 1	0.668	0.721	0.711	0.770	0.739	0.813
EMG 2	0.673	0.679	0.664	0.721	0.696	0.705
EMG 3	0.739	0.743	0.753	0.792	0.761	0.741
EMG 4	0.787	0.788	0.791	0.818	0.790	0.760
EMG 5	0.774	0.798	0.793	0.816	0.793	0.727
EMG 6	0.736	0.737	0.736	0.745	0.729	0.722
EMG 7	0.745	0.772	0.750	0.768	0.779	0.750
EMG 8	0.746	0.797	0.780	0.797	0.788	0.752

a BDSI: Burst Duration Similarity Index.

b EMG: electromyography.

**Table 8.** Statistical results for mode 2 versus mode 3; metric: BDSI^a^.

	Exercise 1	Exercise 2	Exercise 3	Exercise 4	Exercise 5	Exercise 6
EMG^b^ 1	0.569	0.525	0.492	0.567	0.515	0.535
EMG 2	0.605	0.638	0.618	0.565	0.611	0.506
EMG 3	0.686	0.697	0.680	0.718	0.696	0.636
EMG 4	0.740	0.755	0.744	0.727	0.767	0.743
EMG 5	0.760	0.783	0.765	0.783	0.768	0.759
EMG 6	0.623	0.565	0.587	0.584	0.587	0.607
EMG 7	0.717	0.703	0.705	0.642	0.719	0.609
EMG 8	0.692	0.661	0.679	0.657	0.685	0.599

a BDSI: Burst Duration Similarity Index.

b EMG: electromyography.

## Discussion

### Principal Findings

The results of this study demonstrate that the proposed rehabilitation robotic system constitutes an effective tool for task-oriented neurorehabilitation, with advantages over conventional therapy in several physiotherapeutic relevant aspects. First, the previously observed increase in active patient engagement indicates that the system promotes voluntary motor involvement rather than passive movement execution. By dynamically adapting assistance based on task execution quality, compensatory behaviors, and functional errors, the robot facilitates motor learning, a fundamental process for functional recovery. The biomechanical profile observed during intelligent robot-assisted therapy is consistent with the theoretical requirements of neuroplastic motor rehabilitation—the system provides an adjustable assistance that preserves voluntary motor demand rather than replacing it. This is reflected in the pattern of *iEMG* and *T*_*act*_ results, where mode 2 produces at least equivalent muscular engagement to mode 1, while giving the patient greater autonomy over the motion trajectory. From a motor learning standpoint, this configuration aligns with the paradigm, in which task-relevant errors are tolerated up to the threshold of compensation, and assistance is reserved for preventing significant functional or hazardous anatomical compensations [46].

The comparison between control modes 1 and 2 revealed limited statistically significant differences in overall muscular participation, indicating that both modes effectively support active engagement of the musculoskeletal system. However, for some muscular groups and exercises, an intelligent approach outperforms the constant-admittance system. Nevertheless, results suggest that the intelligent control strategy primarily benefits by increasing patients’ volunteer participation and not by increasing total muscle work. The absence of observable statistical *iEMG* differences between modes 1 and 2 at the per-channel level should not be interpreted as a negative effect. The intelligent mode achieves comparable muscular engagement with a fundamentally different control philosophy by allowing voluntary motion and intervening only contextually.

Compared with conventional physiotherapy, robot-assisted training resulted in significantly greater muscular participation across the analyzed muscle groups. This finding indicates that the robotic system can elicit more consistent, task-relevant muscle activation. Moreover, the use of robots reduces therapist-dependent variability and patient passivity that may occur during manual therapy. The higher mean of *iEMG* values observed in mode 2 versus mode 3 requires clinical contextualization. This most likely reflects a combination of 2 mechanisms—the longer robot-aided exercise duration, along with the postural demand imposed by the exoskeleton’s mechanical constraints. This is evidenced by persistently high activation of the latissimus dorsi and pectoralis major across exercises in modes 1 and 2, consistent with their roles in trunk and proximal stabilization. Whether this additional muscular demand is therapeutically beneficial should be verified in longitudinal clinical trials planned as the follow-up to this investigation.

Notably, distinct motion patterns were observed between the different robot control modes. These changes suggest that the robot not only modifies the level of assistance but also influences patients’ movement strategy, which is a critical aspect of recalling motor capabilities. However, joint ROMs during robot-assisted therapy were generally lower than those mobilized in conventional treatment. While reduced ROM could be interpreted as a limitation, it may also reflect a more controlled, functionally relevant movement execution, thereby reducing compensatory strategies. This is particularly the case while comparing mode 1 with mode 2. From a clinical perspective, the reduction in elbow flexion or extension within mode 2 in the context of increased shoulder flexion or extension may be clinically favorable for patients poststroke, in whom pathological shoulder-elbow flexion synergy is a primary recovery obstacle [1]. If the intelligent algorithm’s compensation detection is effectively preventing this pattern, the ROM changes observed here may represent a functionally meaningful impact, which should be verified with the standardized functional assessment within longitudinal trials. In conventional human-provided treatment, this is due to physiotherapists operating closer to the pain threshold.

The *BDSI* values for mode 2 with mode 1 indicate high temporal similarity between the 2 robotic modes. The intelligent algorithm alters assistance intensity contextually but preserves the fundamental activation sequencing established by the constant-admittance reference. On the contrary, analyses of EMG synchronization during exercises confirmed significantly altered muscular activation patterns in EMG 1 and EMG 2 during mode 2 compared with conventional muscular therapy. These changes indicate a redistribution of muscle activity toward more correct motions in terms of their functionality. On the other hand, more significant changes between mode 2 and mode 3 might result from possible motion limitations in robotic devices, which are typical for such structures. Overall, the robotic system proved to be an effective therapeutic tool, reducing compensations and functional errors commonly performed by patients, while maintaining active participation and movement quality.

Nevertheless, several confounding factors should be acknowledged when interpreting the mode 2 versus mode 3 comparison: exercise duration differed systematically between conditions, with physiotherapist-guided sessions being consistently shorter; the level of physical guidance provided by the physiotherapist varied across participants and sessions; the mechanical constraints of the exoskeleton may have restricted some degrees of freedom not constrained during manual therapy; participant heterogeneity may have introduced variability not fully accounted for in the group-level analyses. However, mode 3 and recording of the reference trajectories for mode 1 and mode 2 were performed by the same specialist for all the cases. This aimed to minimize variability from different assistance during treatment from an individual work approach. Despite this, the mentioned factors are acknowledged as limitations and will be addressed in future controlled trials.

### Comparison With Other Works

The obtained results are consistent with and extend previous research on variable admittance control in rehabilitation robotics. Studies comparing variable admittance, which correlated mainly with synergic joint kinematics, with constant admittance have demonstrated increased patient participation, although often with similar EMG effort distributions across conditions [19]. In contrast, our approach to variable admittance, explicitly linked to trajectory deviations, compensatory movements, and functional errors, yielded improved outcomes in selected cases, particularly in movement quality and muscle activation patterns.

Other studies investigating AAN strategies regulated through muscle entropy have reported positive effects primarily in patients with mild impairments (Fugl-Meyer scores between 58 and 65) [21]. In this study, the proposed method was evaluated in a heterogeneous group of participants, and no significant differences in impairment severity were observed, suggesting robustness across varying functional levels.

Furthermore, recent meta-analyses have demonstrated that EMG-based robot-assisted rehabilitation is significantly superior to conventional rehabilitation techniques [47]. Notably, the results obtained in this study are superior, despite the control system not relying on EMG signals for real-time control. This advantage likely arises from prioritizing user intention during treatment, with robotic intervention applied only when hazardous errors or unacceptable compensations occur. This strategy reduces unnecessary assistance and supports autonomous motor control, aligning with contemporary principles of neuroplastic motor rehabilitation.

### Limitations and Planned Continuation

Several limitations of this study should be acknowledged. First, the experimental protocol was limited to a single-session trial, precluding conclusions about long-term therapeutic effects or functional outcome improvements. Therefore, it has also not included a standardized functional pre- or postassessment. Future studies will therefore focus on longitudinal assessments to evaluate sustained motor recovery and gained independence in performing ADLs. This is planned as a follow-up with outcome measures based on the Fugl-Meyer Assessment and Action Research Arm Test.

Second, the relatively small and heterogeneous participant group limits the further applicability and strength of the findings. Future research will focus specifically on neurological patient populations, enabling more targeted analysis of impairment-specific responses to the therapy.

From a technical perspective, limitations related to the mobility of the robotic structure were identified. Planned construction modifications, including removing the developed open bearings, aim to reduce friction and limit the bulkiness of the device. As a result, patients should be able to perform ADLs in almost all natural ROM.

Finally, differences in exercise duration observed in mode 3 highlight the influence of therapist involvement and potential fatigue on the number of repetitions for exercises performed. Future research will investigate how physiotherapist fatigue affects therapy delivery in real-life clinical settings and whether increased treatment dosage or high-intensity protocols can compensate for longer execution times.

### Final Conclusions

The findings of this study confirm that task-oriented rehabilitation can be effectively supported by robotic systems, leading to improved patient engagement, reduced compensatory behaviors, and enhanced movement quality. The proposed intelligent control approach benefits from context-based assessment of task execution, enabling physiotherapists to design individualized exercises that reflect any ADLs. As such, rehabilitation robots have the potential not only to augment conventional therapy but also to provide structured, repeatable, and fully automatic therapy. Thanks to this, the capabilities of physiotherapists can be multiplied, and patients can receive the required care quickly and in higher doses.

## Supplementary material

10.2196/92898 Checklist 1 GUIDED checklist.

## Abbreviations

AAN: assist-as-needed
ADL: activity of daily living
BDSI: Burst Duration Similarity Index
EMG: electromyography
IMU: inertial moment unit
JM: joint motion
ROM: range of motion

## References

[1] Raghavan P (2015). Upper limb motor impairment after stroke. Phys Med Rehabil Clin N Am.

[2] Krug E, Cieza A (2017). Strengthening health systems to provide rehabilitation services. Am J Phys Med Rehabil.

[3] Cornwell L, Doyle H, Stohner M, Hazle C (2021). Work-related musculoskeletal disorders in physical therapists attributable to manual therapy. J Man Manip Ther.

[4] Halford E, Jakubiszak S, Krug K, Umphress A (2024). What is task-oriented training? A scoping review. Scand J Occup Ther.

[5] Banyai AD, Brișan C (2024). Robotics in physical rehabilitation: systematic review. Health Care (Don Mills).

[6] Mashud G, Hasan SK, Alam N (2025). Advances in control techniques for rehabilitation exoskeleton robots: a systematic review. Actuators.

[7] van Westerhuis C, Sanders AF, Aarden JJ (2024). Capabilities for using telemonitoring in physiotherapy treatment: exploratory qualitative study. JMIR Rehabil Assist Technol.

[8] Reicherzer L, Scheermesser M, Kläy A, Duarte JE, Graf ES (2024). Barriers and facilitators to the use of wearable robots as assistive devices: qualitative study with older adults and physiotherapists. JMIR Rehabil Assist Technol.

[9] Jarrassé N, Proietti T, Crocher V (2014). Robotic exoskeletons: a perspective for the rehabilitation of arm coordination in stroke patients. Front Hum Neurosci.

[10] Cao J, Zhang J, Wang C (2024). Variable admittance control of high compatibility exoskeleton based on human–robotic interaction force. Chin J Mech Eng.

[11] He B, Zhou X, Huai Y Admittance control based hemiplegic exoskeleton motion control.

[12] Ranatunga I, Lewis FL, Popa DO, Tousif SM (2017). Adaptive admittance control for human–robot interaction using model reference design and adaptive inverse filtering. IEEE Trans Contr Syst Technol.

[13] Mujica M, Crespo M, Benoussaad M, Junco S, Fourquet JY (2023). Robust variable admittance control for human–robot co-manipulation of objects with unknown load. Robot Comput Integr Manuf.

[14] Yu W, Rosen J, Li X (2011). PID admittance control for an upper limb exoskeleton.

[15] Miller LM, Roldan JR, Rosen J, Kim H, Li Z (2012). Admittance control of an upper limb exoskeleton—reduction of energy exchange.

[16] Aguirre-Ollinger G, Nagarajan U, Goswami A (2016). An admittance shaping controller for exoskeleton assistance of the lower extremities. Auton Robot.

[17] Kim H, Yang W (2021). Variable admittance control based on human–robot collaboration observer using frequency analysis for sensitive and safe interaction. Sensors.

[18] Topini A, Sansom W, Secciani N, Bartalucci L, Ridolfi A, Allotta B (2021). Variable admittance control of a hand exoskeleton for virtual reality-based rehabilitation tasks. Front Neurorobot.

[19] Zarrin RS, Zeiaee A, Langari R (2024). A variable-admittance assist-as-needed controller for upper-limb rehabilitation exoskeletons. IEEE Robot Autom Lett.

[20] Naghavi N, Akbarzadeh A, Tahamipour-Z. SM, Kardan I (2020). Assist-as-needed control of a hip exoskeleton based on a novel strength index. Rob Auton Syst.

[21] Arantes AP, Bressan N, Borges LR, McGibbon CA (2023). Evaluation of a novel real-time adaptive assist-as-needed controller for robot-assisted upper extremity rehabilitation following stroke. PLoS One.

[22] Aurich-Schuler T, Grob F, van Hedel HJA, Labruyère R (2017). Can Lokomat therapy with children and adolescents be improved? An adaptive clinical pilot trial comparing guidance force, path control, and FreeD. J Neuroeng Rehabil.

[23] Lian P, He Y, Ma Y, Liu J, Wu X (2021). Adaptive admittance control of human-exoskeleton system using RNN optimization.

[24] Petrič T, Peternel L, Morimoto J, Babič J (2019). Assistive arm-exoskeleton control based on human muscular manipulability. Front Neurorobot.

[25] Moubarak S, Pham MT, Moreau R, Redarce T (2010). Gravity compensation of an upper extremity exoskeleton.

[26] Zhang L, Yu H, Li D (2025). Reducing upper-limb muscle effort with model-based gravity compensation during robot-assisted movement. Sensors.

[27] Faul F, Erdfelder E, Lang AG, Buchner A (2007). G*Power 3: a flexible statistical power analysis program for the social, behavioral, and biomedical sciences. Behav Res Methods.

[28] Falkowski P, Kołodziejski P, Zawalski K (2026). Measurement-based optimization of a lightweight upper-extremity rehabilitation exoskeleton for task-oriented treatment. Sensors (Basel).

[29] Falkowski P, Zawalski K (2025). Multibody dynamics simulation of upper extremity rehabilitation exoskeleton during task-oriented exercises. Actuators.

[30] Falkowski P, Leczkowski B (2025). The design of 3D-printed open bearings for human assisting robots. J Autom Mobile Rob Intell Syst.

[31] Falkowski P, Pikuliński M, Osiak T (2025). Similarity analysis of upper extremity’s trajectories in activities of daily living for use in an intelligent control system of a rehabilitation exoskeleton. Actuators.

[32] Falkowski P, Pikuliński M, Osiak T, Jeznach K, Zawalski K, Kołodziejski P (2026). Lecture Notes in Networks and Systems.

[33] Sánchez-Moreno J, Moreno-Salinas D, Álvarez-Ortiz JC (2025). Use of inertial measurement units for detection of the support phases in discus throwing. Sensors (Basel).

[34] Konrad P (2006). The ABC of EMG: a practical introduction to kinesiological electromyography. Noraxon Inc.

[35] Stegeman D, Hermens H (2007). Standards for surface electromyography: the European project surface EMG for non-invasive assessment of muscles (SENIAM). Roessingh Research and Development.

[36] EMG signal processing: key techniques and practical recommendations. Noraxon.

[37] Rodriguez-Tapia B, Soto I, Martinez DM, Arballo NC (2020). Myoelectric interfaces and related applications: current state of EMG signal processing—a systematic review. IEEE Access.

[38] Burden A (2010). How should we normalize electromyograms obtained from healthy participants? What we have learned from over 25 years of research. J Electromyogr Kinesiol.

[39] Androwis GJ, Pilkar R, Ramanujam A, Nolan KJ (2018). Electromyography assessment during gait in a robotic exoskeleton for acute stroke. Front Neurol.

[40] Johanson ME, Radtka SA (2006). Amplitude threshold criteria improve surface electrode specificity during walking and functional movements. Gait Posture.

[41] Stojanović M, Andjelković - Apostolović M, Milošević Z, Ignjatović A, Public Health Institute Niš, Center for Informatics and Biostatistics in Health Care, Niš, Serbia, Public Health Institute Niš, Center for Informatics and Biostatistics in Health Care, Niš, Serbia (2018). Parametric versus nonparametric tests in biomedical research. Acta Med Median.

[42] Turner DL, Ramos-Murguialday A, Birbaumer N, Hoffmann U, Luft A (2013). Neurophysiology of robot-mediated training and therapy: a perspective for future use in clinical populations. Front Neurol.

[43] Lee SH, Park G, Cho DY (2020). Comparisons between end-effector and exoskeleton rehabilitation robots regarding upper extremity function among chronic stroke patients with moderate-to-severe upper limb impairment. Sci Rep.

[44] Dias D, Neto MG, Sales S da S (2023). Effect of mobilization with movement on pain, disability, and range of motion in patients with shoulder pain and movement impairment: a systematic review and meta-analysis. J Clin Med.

[45] Hsieh Y wei, Wu C yi, Lin K chung, Yao G, Wu K yuh, Chang Y ju (2012). Dose-response relationship of robot-assisted stroke motor rehabilitation: the impact of initial motor status. Stroke.

[46] Levac D, Driscoll K, Galvez J, Mercado K, O’Neil L (2017). OPTIMAL practice conditions enhance the benefits of gradually increasing error opportunities on retention of a stepping sequence task. Hum Mov Sci.

[47] Huo Y, Wang X, Zhao W, Hu H, Li L (2023). Effects of EMG-based robot for upper extremity rehabilitation on post-stroke patients: a systematic review and meta-analysis. Front Physiol.

